# Epigenetic signature: implications for mitochondrial quality control in human aging

**DOI:** 10.18632/aging.101832

**Published:** 2019-02-20

**Authors:** Patrizia D’Aquila, Alberto Montesanto, Francesco De Rango, Francesco Guarasci, Giuseppe Passarino, Dina Bellizzi

**Affiliations:** 1 Department of Biology, Ecology and Earth Sciences, University of Calabria, 87036 Rende, Italy; * Equal contribution

**Keywords:** methylation, aging biomarkers, epigenetics biomarkers, mitochondrial quality control, geriatric parameters, chronological age, biological, age

## Abstract

Maintenance of functional mitochondria is essential to prevent damage leading to aging and diseases. What is more, the research of biomarkers of aging is focusing on better predicting functional capability along the lifetime beyond chronological age. Aim of this study was to identify novel CpG sites the methylation of which might be correlated to the chronological and biological age. We performed methylation analyses of the CpG sites in candidate genes involved in mitochondrial biogenesis, mitophagy, fusion, and fission, all key quality control mechanisms to ensure maintenance of healthy mitochondria and homeostasis during aging, using DNA samples from two independent datasets composed by 381 and 468 differently-aged individuals, respectively. Twelve potential CpG predictors resulted associated with aging in the discovery dataset. Of these, two sites located within *RAB32* and *RHOT2* genes were replicated in the second dataset. What is more, individuals exhibiting methylation levels of the *RAB32* CpG site higher than 10% were observed more prone to disability than people with lower levels.

These results seem to provide the first evidence that epigenetic modifications of genes involved in mitochondrial quality control occur over time according to the aging decline, and may then represent potential biomarkers of both chronological and biological age.

## Introduction

Research over the past decades largely demonstrated that mitochondria have a crucial role in aging. The progressive production of ROS leads to an accumulation of somatic mitochondrial DNA (mtDNA) mutations and protein modifications with a consequent decline in oxidative phosphorylation (OXPHOS) activity, loss of bioenergetic capacity, and oxidative stress [1–4]. Mammalian cells adopt several systems in order to maintain mitochondrial integrity and function. This mitochondrial quality control includes pathways related to protein folding and degradation as well as systems involved in organelle turnover, shape, and movement [5–7]. An accurate and constant mitochondrial turnover is based on the coordination of biogenesis, occurring by growth and division of pre-existent mitochondria, and mitophagy, namely the self-destruction of damaged/dysfunctional mitochondria via the autophagic pathway. Multiple endogenous and exogenous factors, including nutrients, hormones, exercise, stress, cold exposure, and hypoxia, regulate mitochondrial biogenesis through ubiquitous transcription factors (SP1, YY1, CREB), nuclear respiratory factors (NRF-1, NRF-2) and coactivators (PGC-1α, PGC-1β, PRC) [8–11].In healthy cells, the elimination of damaged mitochondria in mammals by mitophagy is essential and is mediated by a pathway mainly composed of the PTEN-induced putative protein kinase 1 (PINK1) and the E3 ubiquitin ligase Parkin [12–14].

Furthermore, mitochondria are highly mobile and rapidly change their size, shape, and distribution by continuous integration of events of fusion, the joining of two organelles into one, and fission, the division of a single organelle into two. All the above processes, mainly induced by cellular metabolic states, result in the maintenance of a healthy mitochondrial population, pivotal for cell survival and adaption to changing physiological conditions, and thus particularly important to forestall aging [7,15,16].In fact, transgenic mice carrying specific mutations in mitofusin 1 (*MFN1*), mitofusin 2 (*MFN2*) and optic atrophy (*OPA1*) as well as in dynamin-related protein 1 (*DRP1*) and fission 1 (*FIS1*) genes, the master mediators of mitochondrial fusion and fission, respectively, exhibit dramatic decrease in mtDNA content, mitochondrial function swelling, compensatory proliferation, severe fragmentation of the mitochondrial network, suppression of content exchange between mitochondria, and production of interconnected mitochondria [7,16–21]. Several findings highlighted that all the mitochondrial processes above described do not operate independently but they rather influence each other and are subject to concerted regulatory pathways [7].

Deregulation of mitochondrial quality control is one of the intrinsic causes of mitochondrial dysfunction which leads to aging and age-related diseases [22–25]. Several studies demonstrated that mitochondrial fusion is positively associated with the increase of survival in *C. elegans*, and with the exercise-induced longevity in both rodents and worms [26–28]. Conversely, the Mfn2-to-Drp1 ratio, named mitochondrial fusion index, was found significantly increased in muscles of aged rats compared to younger, suggesting an involvement of the fusion in inducing sarcopenia and muscle decay [29]. An age-correlated imbalance in fusion and fission events might increase the mitochondrial mass, if fusion is more prevalent, or mitochondrial number, when fission is more frequent, if extra mitochondria are not eliminated by mitophagy. Mitochondrial biogenesis should occur in order to compensate the decreased mitochondrial biomass resulting from mitochondrial degradation [30]. Consistently, *in vivo* and *in vitro* experiments demonstrated a decline of mitophagy pathway with age and an accumulation in aged cells of enlarged (giant) or highly interconnected mitochondria, with low ATP production, loss of cristae structure and swollen morphology [31].

Here, we aimed to disentangle the correlation between the above processes and aging from an epigenetic point of view. In fact, research identifying biomarkers of aging made extensive use of epigenetic changes of specific loci occurring during lifetime.

To this purpose, we performed a methylation analysis of 1437 CpG sites in candidate genes involved in mitochondrial biogenesis, mitophagy, fusion, and fission in order to evaluate the epigenetic variability of these sites in human aging and aging phenotypes. This analysis was performed by applying the Sequenom EpiTYPER technology to peripheral blood DNAs from two population samples including 381 (discovery dataset) and 468 (replication dataset) differently aged individuals, respectively.

## RESULTS

### CpG methylation profiling of mitochondrial quality control candidate genes in differently aged humans

**Figure 1:**
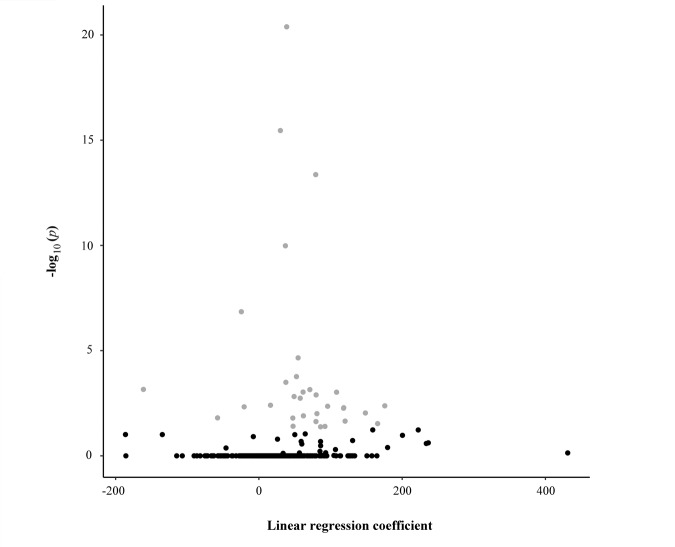
Scatter plot reporting the linear regression coefficients (x-axis) and the corresponding -log10 adjusted p-values (y-axis) of the association between each CpG unit and the age of the study sample. Each dot represents one CpG unit. Grey dots indicate CpG units whose levels were significantly associated with the age of the study sample.

We analysed DNA methylation profiles using Sequenom MassARRAY EpiTYPER, a bisulphite-based technology that relies on base-specific cleavage and mass spectrometry and measures the level of methylation in amplicons containing one or more CpGs. To assess potential epigenetic changes during aging, the profiles were investigated in bisulfite-treated DNA samples collected from 381 subjects (48- to 107-year-old) of the discovery dataset. Specifically, we investigated a total of 1437 CpG sites located within CpG islands falling in candidate genes involved in mitochondrial biogenesis, mitophagy, fusion, and fission processes and annotated in the UCSC genome browser. Following stringent quality control criteria, a total of 500 CpG sites entered further analysis. The sequences of the CpG islands containing these sites and their chromosomal localization are shown in Figure S1. The results of the univariate linear regression analysis are presented in Table 1 and in Figure 1 in which we observed that 54 out of the 500 potential predictors are statistically significant after the Holm method to account for multiple testing. Changes of methylation levels with age at the 54 CpG sites are shown in Figure S2.

**Table 1:** Univariate association analysis between CpG units and age of the sample under study.

**CpG unit**	**T-statistic**	**P-value**	**Adjusted P-value**
BNIP3L_Amplicon1_CpG_10	10.749	1.113*10^-23^	4.090*10^-21^
BNIP3L_Amplicon1_CpG_16.17.18	4.664	4.31335*10^-6^	1.518*10^-3^
COX18_CpG_2	4.702	3.606*10^-6^	1.273*10^-3^
COX18_CpG_9	4.252	2.673*10^-5^	9.194*10^-3^
COX18_CpG_13.14	4.386	1.496*10^-5^	5.176*10^-3^
COX18_CpG_15	4.034	6.625*10^-5^	2.246*10^-2^
COX18_CpG_22.23	4.374	1.580*10^-5^	5.452*10^-3^
GABARAP_Amplicon2_CpG_7.8	3.966	8.758*10^-5^	2.951*10^-2^
GABARAP_Amplicon2_CpG_10	4.774	2.626*10^-6^	9.320*10^-4^
MAP1LC3A_Amplicon2_CpG_16.17	-4.411	1.352*10^-5^	4.692*10^-3^
MAP1LC3B_CpG_10.11	3.894	1.167*10^-4^	3.920*10^-2^
MARCH5_Amplicon1_CpG_2.3.4	-4.835	1.938*10^-6^	6.980*10^-4^
MTERFD1_Amplicon1_CpG_7	4.624	5.201*10^-6^	1.826*10^-3^
MTERFD1_Amplicon1_CpG_16.17	4.998	8.881*10^-7^	3.210*10^-4^
MTERFD1_Amplicon1_CpG_36	5.127	4.744*10^-7^	1.720*10^-4^
MTERFD1_Amplicon1_CpG_39	4.425	1.269*10^-5^	4.415*10^-3^
MTERFD1_Amplicon1_CpG_40	4.436	1.204*10^-5^	4.202*10^-3^
MTFR2_Ampicon1_CpG_9	4.830	1.986*10^-6^	7.130*10^-4^
MTFR2_Amplicon1_CpG_10	4.830	1.986*10^-6^	7.130*10^-4^
MTFR2_Amplicon1_CpG_19	4.830	1.986*10^-6^	7.130*10^-4^
MTFR2_Amplicon1_CpG_30	4.830	1.986*10^-6^	7.130*10^-4^
MTFR2_Amplicon2_CpG_19	3.877	1.253*10^-4^	4.171*10^-2^
MTIF3_Amplicon2_CpG_2	4.451	1.125*10^-5^	3.939*10^-3^
MTIF3_Amplicon2_CpG_16	4.767	2.674*10^-6^	9.470*10^-4^
MTIF3_Amplicon2_CpG_19.20.21	4.024	6.951*10^-5^	2.349*10^-2^
POLG1_Amplicon1_CpG_11.12.13	-6.428	3.896*10^-10^	1.420*10^-7^
POLG2_CpG_1	3.888	1.194*10^-4^	3.999*10^-2^
POLG2_CpG_7	3.888	1.194*10^-4^	3.999*10^-2^
RAB32_Amplicon1_CpG_14.15	4.236	2.880*10^-5^	9.879*10^-3^
RAB32_Amplicon1_CpG_24	4.178	3.669*10^-5^	1.254*10^-2^
RHOT2_Amplicon1_CpG_16	-4.123	4.604*10^-5^	1.570*10^-2^
TFAM_Amplicon1_CpG_3.4	5.527	6.079*10^-8^	2.210*10^-5^
TFB1M_CpG_12.13	8.692	1.183*10^-16^	4.330*10^-14^
TFB1M_CpG_15.16	7.571	2.860*10^-13^	1.040*10^-10^
TFB1M_CpG_31	9.324	9.492*10^-19^	3.480*10^-16^
TFB2M_CpG_38	4.117	4.719*10^-5^	1.600*10^-2^

Starting from the set of 54 CpG units selected by the univariate analysis, we performed a stringent bootstrap-based stepwise selection procedure to remove potential spurious statistical associations. We found that among the most informative CpG markers (markers included in at least 80% of simulated models) there were BNIP3L_Amplicon1_CpG_10 (99.39% times), COX18_CpG_2 (85.16% times), COX18_CpG_15 (81.42% times), GABARAP_Amplicon2_CpG_7.8 (81.42% times), MARCH5_Amplicon1_CpG_2.3.4 (90.76% times), RAB32_Amplicon1_CpG_24 (86.6% times), RHOT2_Amplicon1_CpG_16 (91.31% times) and TFB1M_CpG_12.13 (80.83% times). The model including all these variables explained about 43.06% of age variance of the study sample.

In order to validate our findings, the CpG units selected by the permutation approach in the discovery dataset were tested in the replication dataset. Table 2 reports the results of the association obtained from the test. As it can be observed, we successfully replicated 2 out 8 significant associations and, in particular, those involving RAB32_Amplicon1_CpG_24 and RHOT2_Amplicon1_CpG_16 units.

**Table 2:** Results of the linear regression analysis carried out in the replication dataset.

**CpG unit**	**Beta**	**Standard Error**	**T-statistic**	**P-value**	**Adjusted P-value**
BNIP3L_Amplicon1_CpG_10	9.776	8.671	1.127	0.260	0.999
COX18_CpG_2	0.495	14.754	0.034	0.973	0.999
COX18_CpG_15	-2.961	24.346	-0.122	0.903	0.999
GABARAP_Amplicon2_CpG_7.8	-75.456	53.490	-1.411	0.159	0.954
MARCH5_Amplicon1_CpG_2.3.4	-38.731	54.953	-0.705	0.481	0.999
RAB32_Amplicon1_CpG_24	67.316	23.023	2.924	4.00*10^-3^	2.80*10^-2^
RHOT2_Amplicon1_CpG_16	-122.007	19.275	-6.330	5.94*10^-10^	4.75*10^-9^
TFB1M_CpG_12.13	-9.401	9.502	-0.989	0.323	0.999

### Correlation of methylation profiles with geriatric components and survival at old age

We verified whether the variability of the two validated CpG markers (RAB32_Amplicon1_CpG_24 and RHOT2_Amplicon1_CpG_16) might affect the quality of life in the 60-89 years old group of the discovery dataset. In particular, this age group was analysed for examining the association between the methylation level variability at the analysed units and physical and cognitive abilities measured by geriatric assessments, including Hand Grip (HG), Activity Daily Living (ADL), Mini Mental State Examination (MMSE) and Geriatric Depression Scale (GDS). As shown in Table 3 we found that RAB32_Amplicon1_CpG_24 epigenetic variability was correlated with the risk of disability. In particular, subjects with methylation levels higher that 10% were more than twice as likely to develop disability than people with low methylation levels at that site. A borderline association was also detected between higher methylation level (>10%) at the RHOT2_Amplicon1_CpG_16 and MMSE performances.

**Table 3:** Association between the methylation levels of *RAB32* and *RHOT2* CpG units and physical and cognitive abilities.

**CpG unit**	**HG (SE)**	**P-value**	**ADL OR (95%CI)**	**P-value**	**MMSE B (SE)**	**P-value**	**GDS OR (95%CI)**	**P-value**
RAB32_Amplicon1_CpG_24	-0.465 (0.811)	0.567	2.019 (1.132- 3.601)	0.017	-0.303 (0.542)	0.576	1.240 (0.657-2.340)	0.507
RHOT2_Amplicon1_CpG_16	8.446 (6.863)	0.220	2.348 (0.015-357.763)	0.739	-8.166 (4.653)	0.081	0.053 (0.00-14.926)	0.308

### Expression of *RAB32* and *RHOT2* genes

**Figure 2:**
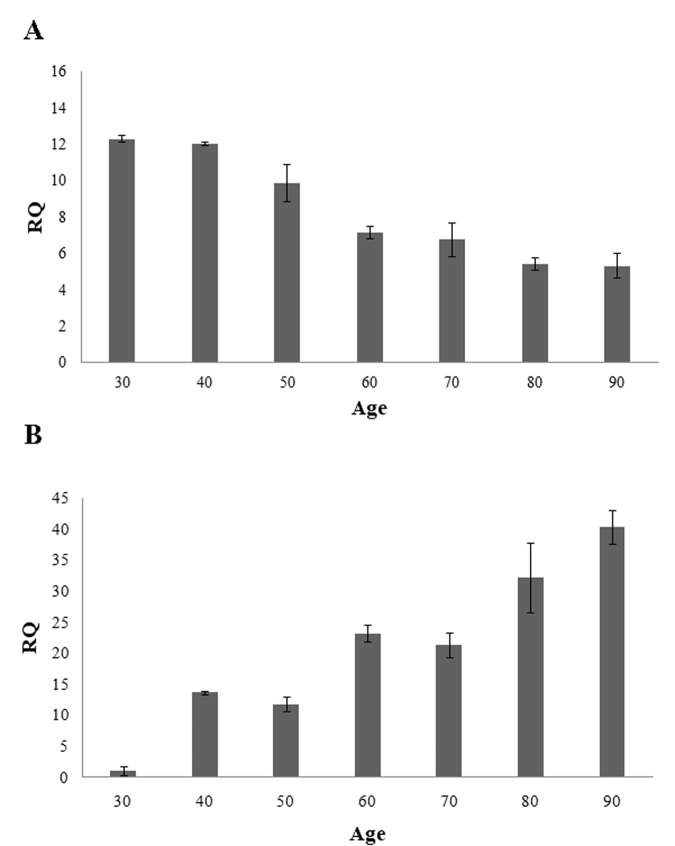
Expression levels of *RAB32* (**A**) and *RHOT2* (**B**) in samples of different ages. These levels are reported as the mean of relative quantification values (RQ), measured in three independent triplicate experiments with standard error mean (SEM).

To explore the functional relevance of the methylation of the CpG islands located within *RAB32* and *RHOT2* genes, quantitative real-time PCR assays were carried out in order to evaluate the expression levels of mRNA in the samples of different ages. Consistently with the methylation patterns above described, we observed that mRNA levels of *RAB32* and *RHOT2* genes decrease and increase, respectively, during age further confirming their potential role as biomarkers of both chronological and biological age (Figure 2A and 2B).

## DISCUSSION

Recent progress in the field of DNA methylation has enabled the identification of numerous genes, and at the same time of a large set of CpG dinucleotides within them, showing a linear correlation of their methylation status with age. These “clock CpGs” can estimate the age of cells, tissues or organs and can predict mortality and time of death [32–37]. In our study, we aimed to reveal novel gene associated CpG sites which exhibit changes in their methylation status during the age. Considering that mitochondrial biogenesis, fusion, fission, and mitophagy contribute to ensuring the quality and the maintenance of healthy mitochondria during age, we explored the methylation levels of candidate genes involved in these processes, so far not investigated. For this purpose, we performed a methylation analysis of DNA samples from whole blood of differently aged human individuals displaying different phenotypes according to cognitive, functional and psychological parameters [38]. Of the 500 sites explored in the univariate linear regression analysis, we identified 54 CpG sites, belonging to 36 genomic regions, differently methylated between young and old subjects from which the highest 12 correlated with the age were selected as significant predictors by very robust bootstrap analysis. Of these 12, two CpGs sites, CpG_24 in *RAB32* and CpG_16 in *RHOT2* genes were confirmed by replication in an independent sample, thus supporting the usefulness of these genes as predictive biomarkers of chronological aging in humans. *RAB32* encodes a GTPase related to the oncogene RAS and plays a role in mitochondrial fission and mitophagy processes and in apoptosis as well. *RHOT2* encodes a protein localized to the outer mitochondrial membrane and is involved in mitochondrial trafficking and fusion-fission dynamics. We detected a strong hyper- and hypo-methylation of *RAB32* and *RHOT2* genes, respectively, with age, in line with the several lines of evidence highlighting the complex nature of the relationship between DNA methylation and aging. In fact, if demethylation was largely described at the global level, hypermethylation and loss of methylation with aging were demonstrated occurring in gene associated CpG sites [39–43]. It is worth mentioning that the analysis of the methylation profile of the two genes in rats of different ages showed the existence of DNA methylation trends in blood similar to humans, but different patterns across heart, liver, kidney and brain tissues (data not shown).

The biological impact of the methylation status of *RAB32* and *RHOT2* genes is highlighted in the expression analysis in which we demonstrated a decrease and an increase, respectively, of mRNA levels of the two genes. It is noteworthy that the *in silico* analysis revealed that CpG_24 in *RAB32* and CpG_16 in *RHOT2* genes overlap with potential c-Jun and E2F-1 binding sites, respectively, which are widely reported being methylation-sensitive transcription factors [44–46]. We are aware that to have not identified the factors that regulate the transcription of the two genes represents a limit of this work. However we believe, in line with different authors, that it is essential the sequence context where a transcription factor binding site is located to determine its functionality [47,48]. As reported in literature, sequences outside the *core* binding site profoundly affect transcription factor binding. In our case, the analysis of a larger sequence would imply the manipulation of neighbouring CpG sites that were not found methylated in the population study, deviating too much from the *in vivo* condition. Therefore, the expression levels of *RAB32* and *RHOT2* genes are regulated during aging by epigenetic mechanisms and it is plausible to hypothesize that they result in regulation of the mitochondrial quality control network, the effects of which, considering the role in this process of multiple molecular components, are not easily predictable.

It is interesting to note that *RAB32* gene is also included in the Horvath’s epigenetic clock, one of the most accurate and precise estimate of chronological age found in the human brain and other solid tissues [33]. This evidence not only confirms the role of this gene as aging biomarker but also that our findings are not specific to blood tissue but are also valid for cells from other tissues. On the contrary, Horvath’s epigenetic clock as well as other epigenetic models for age prediction completely neglected *ROTH2* gene making this gene a potential biomarker useful to improve the prediction accuracy of such model.

It is possible to assume that *RAB32* CpG site is not only a biomarker of chronological but also of biological age since we observed that individuals with methylation levels of this site higher than 10% are twice as likely to be prone to disability with respect to those with lower levels. This result is in line with multiple literature data demonstrating that individuals at the same chronological age may possess dissimilar biomarker signatures reflecting their biological age.

Our study emphasizes the existence of epigenetic regulation of genes coding for components of the mitochondrial quality control, highlighting the role that this control may play during age and the importance of methylated specific CpGs as promising biomarker for chronological and biological age.

## MATERIALS AND METHODS

### Population samples

The discovery dataset included 381 unrelated individuals (185 men end 196 women) aged 48 to 107 years. The subjects older 60 years underwent a thorough geriatric assessment and a structured interview including the administration of a questionnaire validated at European level. The questionnaire collected socio-demographic information, anthropometric measures and a set of the most common tests to assess cognitive functioning, functional activity, physical performance, and depression. In particular, cognitive status was rated by Mini Mental State Examination (MMSE) [49]. Hand grip strength was measured by using a handheld dynamometer (SMEDLEY’s dynamometer TTM) while the subject was sitting with the arm close to his/her body. The test was repeated three times with the stronger hand and the maximum of these values was used in the analyses. The management of activities of daily living (bathing, dressing, eating, independence in and out of bed) was assessed by using the Katz’ Index of activities of daily living [50]. Depressive symptoms were assessed using the 15-item Geriatric Depression Scale [51].

The replication dataset included 468 subjects (206 men and 262 women) aged 18 to 108 years which were recruited at the INRCA Hospital, which is a reference point for the care of the aging people in the Calabria. Also these groups of subjects underwent through a geriatric assessment. Fully informed consent was obtained in writing from all the participants, and all the studies were approved by the Local Ethics Committee.

### DNA samples

Six millilitres of venous blood were drawn from each human subject. Plasma/sera were used for routine laboratory analyses, while DNA was extracted from buffy coats following standard procedures.

Genomic DNA was obtained by phenol/chloroform puriﬁcation. The DNA concentration and purity were determined spectrophotometrically.

### Primer design for EpiTYPER assay

PCR primers for the genes of interest were designed using Sequenom’s EpiDesigner software (Table S1). They do not contain CpGs, amplify both methylated and unmethylated sequences equally, and delimit amplicon of size below 300 bp to increase the amplification success rate, covering as many CpGs as possible.

A T7-promoter tag (cagtaatacgactcactatagggagaaggct) was added to the reverse primers for the *in vitro* T7 transcription and a 10-mer tag sequence (aggaagagag) was added to the forward primers to balance the PCR primer length.

### Bisulﬁte treatment and PCR conditions

Bisulﬁte conversion of each DNA sample was performed using the EZ-96 DNA Methylation-Gold kit (Zymo Research, Euroclone, Milan, Italy), according to the manufacturer’s protocol. Brieﬂy, 1 μg of genomic DNA was added to 130 μl of CT conversion reagent in a ﬁnal volume of 150 μl. The mix was incubated at 98 °C for 10 minutes and, successively, at 64 °C for 2.5 hours. After adding 400 μl of M-binding buffer to the wells of the silicon-A binding plate, each sample was loaded into the wells and centrifuged at 3000 g for 5 minutes. After adding of 400 μl of M-wash buffer to the wells and centrifugation at 3000 g for 5 minutes, 200 μl of M-desulfonation buffer was added to each well and incubated at room temperature for 20 minutes. Then, the solution was removed by centrifugation at 3000 g for 5 minutes and the wells were washed twice with 400 μl of M-wash buffer. Deaminated DNA was eluted in 30 μl of M-elution buffer. The PCRs were carried out in a total volume of 5μl using 1 μl of bisulﬁte-treated DNA, EpiTaq PCR buffer 1X, 0.4 μM of each primer, 0.3 mM dNTP mixture, 2.5 mM of MgCl_2_, and 0.005 U TaKaRa EpiTaq HS (TaKaRa, Diatech Lab Line, Milan, Italy). The thermal proﬁle used for the reaction included a 4 minute heat activation of the enzyme at 95 °C, followed by 45 cycles of denaturation at 94 °C for 20 seconds, annealing at optimal temperature for each primer pair (Table S1) for 30 seconds, extension at 72 °C for 1 minute, then one cycle at 72 °C for minutes. 0.5μl of each PCR product was electrophoresed on 1.5% agarose gel to conﬁrm successful PCR and ampliﬁcation speciﬁcity.

### Dephosphorylation of unincorporated deoxynucleosidetriphosphates, and *in vitro* transcription and RNaseA cleavage

Unincorporated dNTPs in the ampliﬁcation products were dephosphorylated by adding 1.7 μl DNase free water and 0.3 μl (0.5 U) shrimp alkaline phosphatase (SAP) (Sequenom, Inc., San Diego, CA, USA). Each reaction was incubated at 37 °C for 40 minutes, and SAP was then heat inactivated at 85 °C for 5 minutes. Subsequently, samples were incubated at 37 °C for 3 hours with 5 μl of T-cleavage reaction mix (Sequenom), containing 3.21 μl RNAse-free water, 0.89 μl 5X T7 polymerase buffer, 0.22 μl T-cleavage mix, 0.22 μl 100 mM DTT, 0.40 μl T7 RNA polymerase and 0.06 μl RNase A, for concurrent *in vitro* transcription and base-speciﬁc cleavage. The samples of cleaved fragments were then diluted with 20 μl water. Conditioning of the cleavage reaction was carried out by adding 6 mg of clean resin.

### Mass spectrometry

10 nl of the resultant cleavage reactions were spotted onto silicon matrix preloaded chips (Spectro-CHIP; Sequenom) using the MassARRAY nanodispenser (Sequenom) and analysed using the MassARRAY Compact System matrix-assisted laser desorption/ionization-time-of-ﬂight mass spectrometer (MALDI-TOF) (Sequenom). The spectra’s methylation ratios were calculated using EPITYPER software v1.0 (Sequenom). The method yields quantitative results for each of the sequence-deﬁned analytic units referred as CpG units, which may contain either one individual CpG site or an aggregate of CpG sites. Triplicate independent analyses from sodium bisulﬁte-treated DNA samples were undertaken. The effectiveness of the entire experimental procedure was assessed by analyzing as control CpGenome Universal Unmethylated DNA (Chemicon) and CpGenome Universal Methylated DNA (Chemicon, Millipore, Germany) in serial mixtures of methylated and unmethylated products, with 10% methylation increments. Data quality control and ﬁltering were carried out by the removal of the CpG dinucleotides whose the measurement success rate was <90%. Poor-quality and non-valuable data for the quantitative methylation of each CpG unit measured by MALDI-TOF-MS were excluded.

### Expression profile analysis of the *RAB32* and *RHOT2* genes

The total RNA was extracted from the blood of individuals of various age (30–90 years old) using ReliaPrep RNA Tissue Miniprep System (Promega Corp, Italy). Briefly, 2.5 ml of fresh whole blood was mixed to 7.5 ml of RNA red blood cell lysis solution and incubated at room temperature for 10 minutes. White cells were isolated by centrifugating samples at 3000 g for 10 minutes and lysed in 200 μl of LBA+TG buffer and 85% μl of isopropanol. The lysate was then transferred to a ReliaPrep minicolumn, and RNA was purified according to manufacturer's recommendation.

The RNA concentration was measured for each sample using a spectrophotometer and purity of the sample was evaluated using the 260/280 nm absorbance ratio. RNA samples were treated with DNA‐free DNase to remove any residual genomic DNA contamination.

Reverse transcriptase‐PCRs (RT–PCR) were carried out using the RevertAid RT Kit (Thermo Fisher Scientific, Milan, Italy). First, a RT mix including 500 ng of total RNA and 1 μl of Oligo(dT)18 primers was preheated at 65 °C for 5 minutes. Then, the reaction was carried out in a 20 μl final volume containing 1X reaction buffer, 20 U of RiboLock RNase inhibitor, 1 mm of dNTP mix, and 200 U of RevertAid M‐MuLV RT reverse transcriptase. The mix was incubated at at 42 °C for 60 minutes and, successively, at 70 °C for 5 minutes to inactivate the reverse transcriptase. The cDNAs obtained were then used as a template for real‐time PCRs carried out using the SYBR Green qPCR Master Mix (Promega) in a StepOne Plus machine (Applied Biosystems, Milan, Italy).

The final PCR mixture (10 μl) contained 1μl of cDNA, 1X GoTaq qPCR Master Mix, 0.2 μmoles of each primer, and 1X CXR reference dye. Forward and reverse primers were as follows: RAB32For 5’-CAGGTGGACCAATTCTGCAAA-3’; RAB32Rev 5’-GGCAGCTTCCTCTATGTTTATGT-3’;

RHOT2For 5’-TGGAGCTGACTGCGGACTAT-3’; RHOT2Rev 5’-TCTGCACAAACTGGTAGC

CAA-3’; GAPDHFor 5’-ATGGGGAAGGTGAAGGTCG-3’; GAPDHRev 5’-GGGGTCATTGAT

GGCAACAATA-3’. The thermal profile used for the reaction included a 2 minutes heat activation of the enzyme at 95 °C, followed by 35 cycles of denaturation at 95 °C for 15 seconds and annealing/extension at 60 °C for 60 seconds, followed by melt analysis ramping at 60–95 °C. All measurements were taken in the log phase of amplification. Negative controls (in which water instead of cDNA was added) were also run in each plate. StepOne Software V 2.0 was used to analyze data. Gene expression values were normalized to GAPDH gene expression, used as internal control. In addition, the normalized values measured in the 30‐year‐old human were used as reference values (relative quantification) for the other samples.

### Statistical analyses

Linear regression analyses were carried out to evaluate the level of DNA methylation as a function of age. To evaluate the combined effect of the analysed CpG markers with respect to the age of sample under study, we used a stepwise variable selection procedure based on the Akaike Information Criterion (AIC) combined with a bootstrap re-sampling method. In brief, this algorithm firstly simulates a new dataset taking a sample with replacement from the original dataset; secondly, it runs a stepwise selection procedure based on AIC on this simulated dataset, and, finally, repeats the previous steps *n* times. At the end of this procedure, this algorithm records how many times each variable (i) was selected, (ii) the estimate of the regression coefficient was statistically significant, and (iii) changed signs. The final model was selected by retrieving the variables that were selected in at least 80% of the bootstrap samples using linear regression. The prediction accuracy of the developed prediction model was assessed using the adjusted R^2^ parameter, which is a measure of the proportion of age variance explained by a particular set of CpG predictors and their combined effect. Statistical analyses were performed using the R statistical language program (http://www.R-project.org/). In particular, the CpGassoc package was used to perform a linear regression analysis using the Holm method to account for multiple testing; the bootStepAIC package was used to perform stepwise variable selection procedure combined with a bootstrap resampling approach and, finally, *ggplot2* packages were used for graphics purpose [52].

Student’s t-test was adopted to compare methylation proﬁles with respect to the analysed geriatric parameters.

## Supplementary Material

Supplementary Figures

Supplementary Table S1
