# Supplementary material for: Epigenetic signature: implications for mitochondrial quality control in human aging
Source: Aging (Albany NY). 2019 Feb 20;11(4):1240–51. doi: 10.18632/aging.101832 (PMC6402507; doi:10.18632/aging.101832)
Supplement: Supplementary Figures [file aging-11-101832-s001.docx]

**SUPPLEMENTARY FIGURES**

**BNIP3L_Amplicon 1 (Chromosome 8)**

GGCTTGTTGT GTTGCTGCCT GAGTGC**CG**GA GA**CG**GTCCTG CTGCTGC**CG**C 26383109

AGTCCTGCCA GCTGTC**CG**AC AATGT**CG**TCC CACCTAGT**CG** AGC**CG**C**CG**C**C** 26383159

**G**CCCCTGCAC AACAACAACA ACAACTG**CG**A GGAAAATGAG CAGTCTCTGC 26383209

CCC**CG**C**CG**GC **CG**GCCTCAAC AGTGAGTG**CG** GGGC**CG**AGGC TCTGTGAAGG 26383259

GGATGGGGGA GGAGGAGCAG CCC**CG**GC**CG**C **CG**CCAC**CG**G**C GCG**G**CGCG**GG 26383309

AGG**CG**GGAGG AGAAGGCAGC TCATTGGCTC


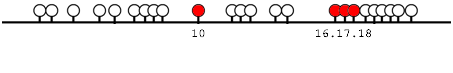


**COX18 (Chromosome 4)**

GGATGTAGTG CTGGTAGGCT GCCAAAGGCA G**CG**TGACAGC ACCC**CG**TAAG 73069374

GCCA**CG**GTGG AGAGCAGAAT GCTGCCCCAC CAGGGCAGGC C**CG**TGG**CG**G**C** 73069424

**G**TGCA**CG**C**CG** AGCAGTACTT CCTC**CG**CAAC C**CG**CAC**CG**G**C G**AAGA**CGCG**G 73069474

CCAGGGCCT**C G**TACCAGC**CG** TT**CG**CATGTA CTGCAGAGAC TGGTGCCACT 73069524

GCCCACACTG GGAGAGTGGG G**CG**CTTGG**CG** C**CG**CT**CG**TAG GAAC**CG**G**CG**C 73069574

AAG**CG**GCAGG TCCCTAGCCC AAAGCTGCAG GGC


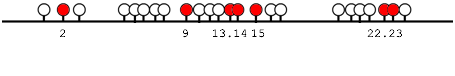


**GABARAP_Amplicon2 (Chromosome 17)**

GTCCCCTCAA GGAAGCTGGG GCTGAAGAGG AGTAAAAGAT GGTAATCATC 7242731

ATA**CG**AGACT TGGTTCTCCA AGTTCCTTT**C G**TTAACAA**CG** TAGAGGAACA 7242781

GCAGGGACAA TTACAAGGTT AGCTATTCA**C G**AAC**CG**TGTT GCTA**CG**CTGA 7242831

AGG**CG**GC**CG**T TGACACCAAA ACAAAGTAGT ACCCAAGTGG **CG**GAGATGAT 7242881

CTCCAGAAAT AAGAAGTCAA AAAGAAAACA GATGTTTGGA GAGATCTACA 7242931

GAA**CG**CTTAA GTGCCAAAAT GAGTAGACCA ATAGGGACTG GAGAGGAGGT 7242981

TGGAAAGGTA GGGACTA**CG**C CAG**CGCG**GAG GAACACTG**CG** GGACTTGAGT 7243031

TAAATCATGT GATCTC


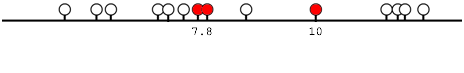


**MAP1LC3A_Amplicon2 (Chromosome 20)**

GCTGTGGGGC CTGATGGCCC **CG**GGGGTGGG GGCTGGAGCT GGGG**CG**TGGC 34559128

**CG**GGGGC**CG**C CCCTC**CG**GGA CAGG**CG**GGG**C G**GACCTGGGC **CG**GCC**CG**ACC 34559178

**CG**GCCTCA**CG** GTCTGGC**CG**C TGTC**CG**CAGC **CG**AC**CG**CTGT AAGGAGGTAC 34559228

AGCAGATC**CGCG**ACCAGCAC CCCAGCAAAA TCC**CG**GTGAG TCC**CG**CACCC 34559278

CCAGCCCTGC CC**CG**CCCC**CG** CCT**CGCGCG**T TCC**CG**ACA**CG** ACCCCCTGCC 34559328

**CG**CC**CG**CCCT GCTCCCAGGT GATCAT**CG**AG **CG**CTACAAGG GTGAGAAGCA 34559378

GCTGCC**CG**TC CTGGACAAGA CCAAGTTTTT GGTCC


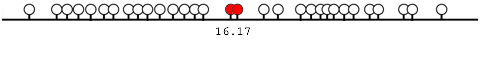


**MAP1LC3B (Chromosome 16)**

GATGTGGGGC AGGCCTGGCA GT**CG**CCACAG A**CG**ACCTAA**C G**GTAGGAAAA 87391589

TCTTACAGCC ACCAGGAGAG TTCCAGG**CG**C **CGCG**GCAGGG GGACTGGGAG 87391639

AGGGGACTG**C G**CCCAGAATG AAGGCT**CG**GG ACAAAAGCAG TTG**CG**CAAA**C** 87391689

**GCG**CCAAGGC TGGG**CG**T**CG**A GTGAC**CGCG**G G**CG**GAGGTCA CCAG**CG**GCCA 87391739

CTCCC**CG**GAA GCCACCCA**CG** GACCA**CGCGC G**CCCCTGCA**C G**CAGAGGGGG 87391789

CCAGGGCTCC A**CG**GG**CG**AG**C G**G**CG**ACCCTG CCTCC**CG**GAG A**CG**G**CGCG**GC 87391839

CTGCCCTG**CGCG**CCTCAGCC C**CG**GGTGC**CG** G**CG**TCT**CG**GG CAGCACCACC 87391889

AAGTCTCTCT GGAGGGGAAA G


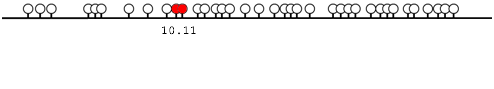


**MARCH5_Ampicon1 (Chromosome 10)**

AGGTGGTGTA ATTCCCCCAA AATGGGCTCT GC**CG**CAGGAG AGGCTGGCTC 92290743

CAG**CG**C**CG**GG GGCTT**CG**GAA GGAGTTTCTG CCACCCCCAC TGC**CG**CCACT 92290793

GAC**CG**CCC**CG** A**CG**CCA**CG**GC **CG**GGGC**CG**GG GACCCTGATA AGAAATGGCC 92290843

CTTCAGCCCC CTCCCCTCAC CTGGCT**CG**GT CCCACCTGAG GGCAAGAG**CG** 92290893

GAGGCAAAAA CAAACAGGCA GGGAGGGCTG A

**
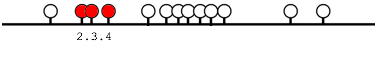
**

**MTERFD1_Amplicon1 (Chromosome 8)**

AACCTAAAGG CCCTTGAGGC CCTGGGG**CGC G**GTCTGGA**CGCG**GGTG**CG**C**C** 96261443

**G**GAACCTGAA CCCT**CG**CCAG GAG**CGCG**ATC CT**CG**TGCTGG GGCAGCTCCT 96261493

GCTTTACCTG TGG**CG**AGGCC TGCTTCC**CG**T AG**CG**GGTGAC CC**CG**GGAC**CG** 96261543

ACCAACT**CG**C TGGGC**CG**CA**C G**TCC**CG**TCC**C G**C**CGCG**C**CG**C A**CG**C**CG**GCTC 96261593

CTCAGCC**CG**C CCTACACAG**C G**CAGC**CGCG**C TCC**CG**GGCCC A**CG**TGGGC**CG** 96261643

**CG**CC**CG**GAGT GGG**CG**AGACC ATGTGC**CG**GG TTA**CGCG**GGG AGACAG**CG**GG 96261693

CTA**CG**TGCTC TGAAAAGAGC CAGCCAGGGG CCC**CG**GCTAC CCTTCTC**CG**G 96261743

CT**CG**CTATTC CTCTGAAGTC CTGTGGACTT GGAGTCTGAA AATTCCT**CG**A 96261793

GTGGTAGAGA TGCCTCAGCT AGGATGA


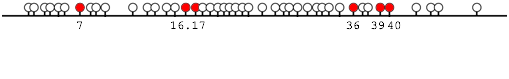


**MTFR2_Amplicon1 (Chromosome 6)**

GGTCACAAGC TTCCTGGTGC TCTCCAAACA CAG**CG**TGGTA GCCTTGCTCA 136249974

CCTTCCTC**CG** AAGC**CG**AGCC CC**CG**GGAATC ACCAGAACAT CAAG**CG**GAGG 136250024

G**CG**TTAGGAA TCCTCAATCA AATTCCAGAC TG**CG**CCACTC CTTGTGCAGA 136250074

T**CG**CTCAGA**C G**TCTACCCAG ACCT**CG**ACCC AGTTTCCACC **CGCGCG**CCT**C** 136250124

**G**GCTTGCAGC CACAGG**CG**GT AA**CG**ATGCCC TCAG**CG**AGGA ATAAGAGGTC 136250174

AACC**CG**GTGC CCAGCCCTGG AGCCTCA**CG**C TCAGCCAGGG TAATGTTATG 136250224

GGAAG**CGCG**C CCC**CG**TCCTC CTG**CG**CCCAA CCAACCCACC TGCTAGTCCC 136250274

TAGGAC**CG**GA CTGCTACTTC **CG**CATGACAG GACTGCTACT TC**CG**CATCCC 136250324

AGGA**CG**AAAT CAAACTC**CGC G**GGCCAGGAC C**CG**TAACCAG CCTCATTGGG 136250374

CAAAACTGAA GAT**CG**CTGCT TCTGATTGGT CATTGCATGA TGTCAGGCAA


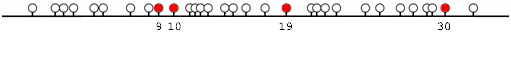


**MTFR2_Amplicon2 (Chromosome 6)**

TGCTTCTGAT TGGTCATTGC ATGATGTCAG GCAATT**CG**GG AAG**CG**ACTGC 136250440

TTTTCCTTCT CAAGAGG**CG**G GTCTTC**CG**AG AGTCAGCCAA TAGGAGCTGA 136250490

**CG**GGCAGGGC AGGT**CGCG**TT AGGACAG**CG**T **CG**CTCC**CG**CA GGGCAAGGTT 136250540

TTAGGTACAA ACT**CG**TGAGC TGGACT**CG**CA GTC**CG**GAGTT **CG**TACCCCAC 136250590

AGAACTCTT**C G**CAGGGAGTG TCTTACT**CG**C TACAGC**CG**GA CCCTAAG**CG**C 136250640

ATC**CG**CCTTC CCAAGTTCAT CTGACACAAA AT**CGCG**ATTT ATTTTTTGGT 136250690

CATTTCATTT GTTCAGCTAA TATTAAAATA CTTGTGGAAC TGGCAGAAGC 136250740

CAAG


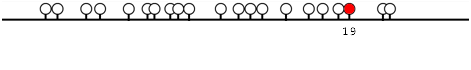


**MTIF3_Amplicon2 (Chromosome 13)**

AACTAGAAAA GCTCAGTGGG GCTGG**CG**GCA ACTCTAGGTC TAACATCCCT 27450291

**CG**GAAGTT**CG** TGCAG**CG**GCC T**CG**CTGAAAA TGGCTTTACA GCC**CG**GCAGA 27450341

ACCCTC**CG**AC TC**CG**CAGCAG GACCTG**CG**GA GCCTCTC**CG**C CCT**CG**GTAC**C** 27450391

**G**G**CG**GGA**CG**G GGTAGCCCTG ACCTTC**CG**GG TGCCTCCTCC ACAGGGG**CG**C 27450441

CCCCTAAGGC CA**CG**CCCTCC **CG**ATGC**CG**GT C**CGCG**CAC**CG** CTC**CG**CA**CG**C 27450491

CTCATATTTA GCATTACCTG TGCTGGGGCA AG**CG**ATTGAC ATACTGTAG**C** 27450541

**G**GA**CG**CAAGT ACAG**CG**GATC TG**CG**G**CG**AGT CCCCTT**CG**CT CTC**CG**TAGTG 27450591

GG**CG**GGGCTT CACCC**CGCG**T CCTTTAAAGG AAAGGTGATG GG**CG**GAGCCA 27450641

**CG**TTTTTCTA CTGTTGGGAT TCAGTTC**CG**C **CG**ACAGAAAA TG**CG**GAGAAC 27450691

G**CG**GAGGAAG TCCCTGAGGG GAAATTGCTG CTCACCTGGC TCC


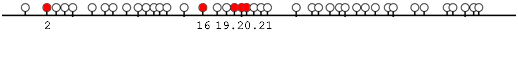


**POLG1_Amplicon1 (Chromosome 15)**

GGGGCAGCTG GGCCTGCAAC AGCAAGTTGG C**CG**CCTCCAG GTAGGGCAGG 89333314

CTCTGCTTCT GGGCCAGGAG G**CG**GAAGTGC TGGTCCAGGT TGTCCC**CG**TA 89333364

GAGGGG**CG**GC AGG**CG**CAGCT CCA**CG**T**CG**GG CAAGGGCA**CG** GCTGGCTGCC 89333414

CCCAGAGCC**C G**TGCTTCTGC AGGTGCT**CG**A **CG**CTG**CG**G**CG** CAC**CGCG**GCC 89333464

T**CG**CCAGGCA TCTCCCCTCC TTGCC**CG**AAG ATTTGCT**CG**T GCAGCCCTCT 89333514

**CG**AGAGCATC TGGATGTCCA ATGGGTTGTG C**CG**CAGCTGC C**CG**CCCTC**CG** 89333564

AGGATAGCAC TTG**CG**GCTGC TGAGGCTGCT GTTGCTGCTG CTGCTGCTGC


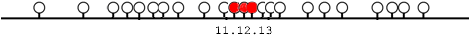


**POLG2 (Chromosome 17)**

AACAGGCACC TGCAGACCTT ATGGCAGGCC CTGA**CG**GCTA CA**CG**AGAG**CG** 64496965

CATCTCTCTC **CG**AAGTTAAA GAGCACACTC TCCCATCACT CAA**CG**GATCC 64497015

CAACAAGCCA CCACTAC**CG**T TAACAGAATC **CG**GAGAGGCC A**CG**G**CG**CAGG 64497065

**CG**CAA**CG**GAG GTGAG**CG**TGC TTG**CG**GG**CG**G CAGGCCCCAC CC**CG**GAAG**CG** 64497115

CATGTCTG**CG** TTC**CG**GC**CG**C AGC**CG**TCCCC **CG**CCCACCAT GG**CG**GA**CG**CC 64497165

GGCT**CG**GATG GTTCTCTGCT AGCTTG

**
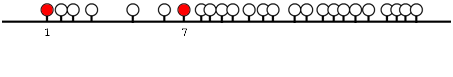
**

**RAB32_Amplicon1 (Chromosome 6)**

GGGAGAGGAA GTCCAGCTGG GCC**CG**GC**CG**G GCTT**CG**GAGG **CG**C**CG**CC**CG**G 146543563

GAGAGGAAGT CCAGTTGGGC C**CG**GC**CG**GGC TTCAGAGG**CG** CAGGG**CG**GGA 146543613

GC**CG**CCT**CGC G**CAGGGTCCT CCCCAAGC**CG** G**CG**CCAGGCC CTGCCCT**CG**T 146543663

C**CG**GCCCTGC CCT**CG**TCTGG CCC**CG**CC**CG**G GC**CGCG**AGCA CTGG**CG**GGTT 146543713

CTGGGTCCTG TGAC**CG**GTCA GG**CG**G**CG**TCA G**CG**GG**CGCG**G **CG**GAGGGCTG 146543763

GC**CG**GCCT**CG** GGGGAGTTTC **CGCG**GC**CG**CC GGGGG**CGCG**G **CG**GCAGAG**CG** 146543813

**CG**AGGC**CG**GG CAGGGGGCCA GACT**CG**GAGT **CG**AGG**CGCG**C C**CG**ACAGC**CG** 146543863

CAG**CG**CTCAT GG**CG**GG**CG**GA GGAGC**CG**GGG ACCC**CG**GCCT GGGGG**CG**GC**C** 146543913

**G**C**CG**CCCCAG **CG**CC**CG**AGAC C**CGCG**AGCAC CTCTTCAAGG TGCTGGTGA

**
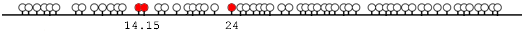
**

**RHOT2_Amplicon1 (Chromosome 16)**

TGGAGTCTCT TTGTCCCCCT AGAAGC**CG**AG CAGA**CG**GA**CG** AGGAGCTG**CG** 668683

GGAGGAGATC CACAAGGTAC C**CG**TGGTG**CGCG**GGA**CG**AGG GAGGGGCTGG 668733

G**CGCG**GGCT**C G**GCCTAATC**C G**CTT**CG**CAGC CTGGGGGATT GGAC**CG**AGGT 668783

GCTC**CG**GGTG TCCTTGGCCC TGATAATTCT GTGACCTC**CG** CACTGAGGGT 668833

TGT**CG**GGGCC CCTACAG**CG**C ACCC**CG**CTGG GAGC**CG**GCAC **CG**CTCAGTCC 668883

AGTGGTGCTC CAGGGATAAC AGGACCC

**
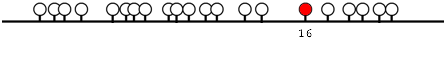
**

**TFAM_Amplicon1 (Chromosome 10)**

GGGGTCCTGG ATGCAGGACT GTCTGTTA**CG** TACAGCCCTT GTGAC**CG**TCA 58385115

**CG**GG**CG**GACA C**CG**GCCAA**CG** C**CG**GGTTGGG GTGAGGC**CG**C **CG**C**CGCG**GTC 58385165

CCTCCATCAC CCTCCTGGCC **CG**GCAGAGGA ACCCACTGCT C**CG**GG**CG**GC**C**  58385215

**G**GGGACAGAG GTGGCTCAAC AG

**
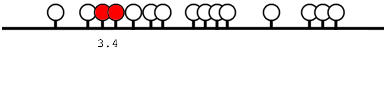
**

**TFB1M (Chromosome 6)**

CCTCAAGTCC AGGAGGAAAT TCTGTGATAG CTGCTT**CG**CT GCTTGCAGTC 155314352

TTAACAACTT AATGATTTCT **CG**AAT**CG**TGG GCAA**CG**GAGG GAGA**CG**GCAA 155314402

GTGCTGAGTT TTC**CG**GAGGC AGCCATGATA **CGCG**GCAAGC ACCATCCAAC 155314452

CCTACCTCAC CCAGGACCTT CAC**CG**C**CG**CT C**CG**AAAGAAA **CGCG**CAGGGG 155314502

AGGAACCTG**C G**AGACCTAAG GCC**CG**CCT**CG** GAGTCAGCCC CATTGGTCAG 155314552

ACCTATCCCA C**CG**GAAG**CG**A TGAC**CGCG**GA CAGGAAATTC C**CG**G**CG**TGCT 155314602

GAGAG**CG**CAT G**CG**CTAAGTC CTGC**CGCG**AG AAGGGCAGGC TGGGTGGT**CG** 155314652

G**CG**GCTTC**CG** CCTGTGAGAG C**CG**GGGGAGA GC**CG**GGTGGA CTAGGCTTCT 155314702

CCTGG

**
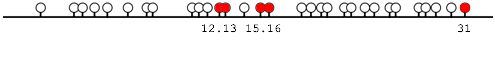
**

**TFB2M (Chromosome 1)**

TGGGATCCAC ATGTCCTTGT CTCTCAGGCC **CG**CTCCAAGA ATCACCTAGT 246566176

GCAGCTACTA CAGTGAACCC CA**CG**CAGGGT ATCCCA**CG**TG GAACATTTTC 246566226

TGG**CG**TC**CG**G GCCAGGTCAA G**CG**GAAGTAA ACACTAGAGC CTG**CG**CATG**C** 246566276

GAACAG**CG**GA GCCTTCCTGC TTTTCTCCCT CACTTC**CG**CT TC**CG**CCT**CG**G 246566326

CTCAGC**CG**CC **CG**AGGATTGT GAGTGGAC**CG** TTGAGGAGAG **CG**AC**CG**ACCA 246566376

TC**CG**GCTGGT GTC**CG**GACT**C G**TACTCTATG GTTGTC**CGCG** CTCTG**CG**CTT 246566426

CCTCTCTAGC **CG**CCAGTGCT CTATGCTC**CGCG**GT**CGCG**GG C**CG**CCAGCCT 246566476

CCAGC**CG**GCC AGC**CGCG**AGG GGTG**CG**CAGA GGGAGG**CG**GG G**CG**GAAAGG**C** 246566526

**G**AGAGGTGTC TCCTCCAC**CG** GAGCCAGGGG AGACC**CG**AGC AAGCTC**CG**TG 246566576

ACAGCA**CG**T**C G**GC**CG**CCATG T**CG**C**CG**AGTG GGGCTGGAAA CAGACC 496


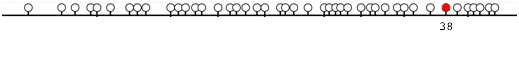


**Figure S1.** Localization of the 500 CpG sites (red) which passed quality control criteria. In particular, the 54 sites statistically significant after the Holm method are underlined. The organization of the sequence-analytic units performed by Sequenom software is also shown.


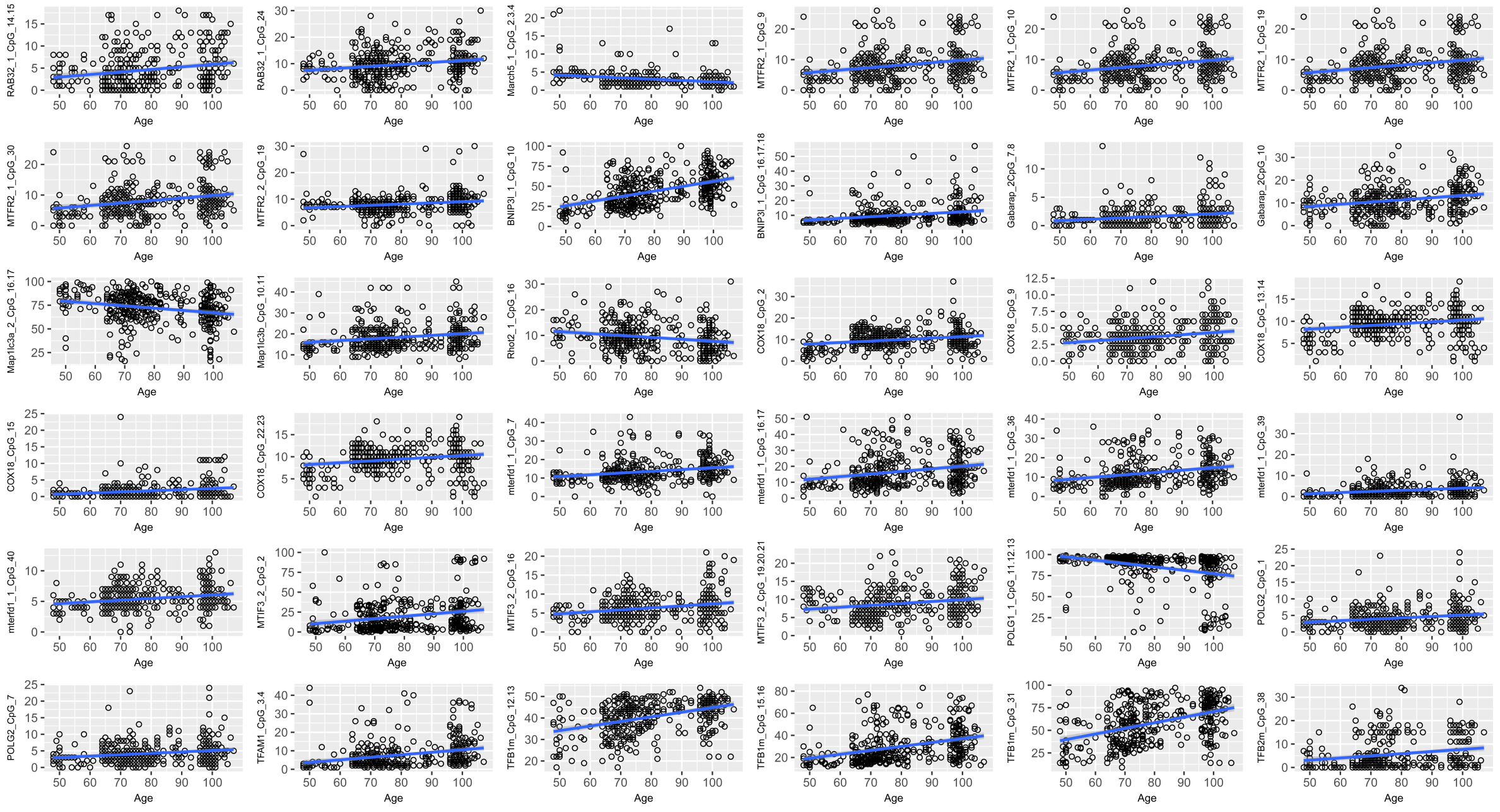


**Figure S2.** Scatter plot of DNA methylation values of the 54 CpG sites statistically significant after the Holm method as a function of human age. For each the blue straight line represents least‐squares linear regression line.
