# Supplementary material for: Epigenetic signature: implications for mitochondrial quality control in human aging
Source: Aging (Albany NY). 2019 Feb 20;11(4):1240–51. doi: 10.18632/aging.101832 (PMC6402507; doi:10.18632/aging.101832)
Supplement: Supplementary Table S1 [file aging-11-101832-s002.docx]

**SUPPLEMENTARY TABLE**

**Table S1. Designed PCR primers of the amplicons analyzed in the study.**

| **GENE** | **Target lenght (bp)** | **Primer sequences (5’-3’)**  **Forward Reverse** | | **Annealing (°C)** |
| --- | --- | --- | --- | --- |
| BNIB3L_Amplicon1 | 280 | AGGGTTTGTTGTGTTGTTGTTTGAGT | AAACCAATAAACTACCTTCTCCTCC | 62 |
| BNIB3L_Amplicon2 | 179 | AGATGGGGGAGGAGGAGTAGTTT | ACCCAAAACCCCAAAAAACA | 62 |
| BNIP3_Amplicon1 | 175 | AGGGATTTGTAGGTAGTTGGAGTT | AAATTTTTATTTTTCTATTTTATTTATTCT | 58 |
| BNIP3_Amplicon2 | 275 | GTTTTTTTGGTTTTTTTTAGGTTTT | CCCTACCCTATAAATTCCTCC | 60 |
| COX10_Amplicon1 | 363 | AGGGAAGTTTTTTAGGTTTTAGATTTGTT | CCAAAAACTCCCCAAAATTACA | 60 |
| COX10_Amplicon2 | 229 | TTGTGTTGGATATTATTATATGGATTT | TCCCAATACTCTCAACTAAAAAAAA | 56 |
| COX18 | 288 | GATGTAGTGTTGGTAGGTTGTTAAA | TCCCTACCCTACAACTTTAAACTAAAAA | 60 |
| DNM1L | 365 | GTTTGTGGGAGGAGGGTTTTG | CAATCCCTAAACAAAACAAAAAAAA | 58 |
| ENDOG_Amplicon1 | 166 | GGAGTTAGGTTTGATGATGGTGGTTAAT | TCCACTCACAAACACTACCAAAAA | 58 |
| ENDOG_Amplicon2 | 476 | GGTTGGGTTTTGTTGTTTTTTTT | CAACTACTCCACCACCCAAAAC | 60 |
| FIS1 | 185 | AGGGTTTTTATTTGATTTTTTTTAGGA | CCCCTACCACTAAACCATAA | 60 |
| GABARAP_Amplicon1 | 456 | GTTGGATAGGGTTGGGTTGAG | AACCTTATAATTATCCCTACTATTCCTC | 60 |
| GABARAP_Amplicon2 | 366 | AGGTTTTTTTAAGGAAGTTGGGGTTG | AAAATCACATAATTTAACTCAAATCCC | 58 |
| KIF5B | 306 | AGGGGAGAGTGGTTATTTTTTTT | CCTACCTCCCCAAAAACTTCTAC | 60 |
| MAP1LC3A_Amplicon1 | 414 | AGTTTTTTTTAAGGAATGTTGTGATTT | AACCATCAAACCCCACAACC | 58 |
| MAP1LC3A_Amplicon2 | 335 | GTTGTGGGGTTTGATGGTTT | AAACCAAAAACTTAATCTTATCCAAAA | 58 |
| MAP1LC3B | 371 | GATGTGGGGTAGGTTTGGTAGT | CTTTCCCCTCCAAAAAAACTTAATA | 60 |
| MARCH5_Amplicon1 | 231 | GTGGTGTAATTTTTTTAAAATGG | TCAACCCTCCCTACCTATTTATTTT | 60 |
| MARCH5_Amplicon2 | 385 | GGGTTGTGTAGTTTTTAGTGGAG | AACCAAACCCAAAAACAATAACACT | 60 |
| MARCH5_Amplicon3 | 106 | TAGTGTTATTGTTTTTGGGTTTGGT | ATCCAACATCTACTATAAAACTTAATCC | 60 |
| MFN1 | 202 | GTTTTGTGGGAAAGGAGAGAGTTAG | AACACCTACCTTAAAAAAAACCTCC | 60 |
| MFN2 | 187 | AGGGAATTATAGTTTTTATGATGTAGTGGGA | AAACTAATAAACCCTAAACCCAACC | 60 |
| MTERF | 154 | GGTTTGGTAGGGGGTAGTAAGAGA | CCCCCTATAAAATCCCTATAAAATACC | 54 |
| MTERFD1_Amplicon1 | 427 | AATTTAAAGGTTTTTGAGGTTTTGG | TCATCCTAACTAAAACATCTCTACCA | 60 |
| MTERFD1_Amplicon2 | 226 | TTTTTTGTTTTTAGTTTTTGTTGGG | TAAAACAACCAACCCACTTCCT | 60 |
| MTFR1 | 404 | AGGAGTTTGAGGAAATAGTTGATTGA | AATCTCAACCTTCAAAACAAATC | 60 |
| MTFR2_Amplicon1 | 500 | GGTTATAAGTTTTTTGGTGTTTTTT | TTACCTAACATCATACAATAACCAATC | 60 |
| MTFR2_Amplicon2 | 354 | TGTTTTTGATTGGTTATTGTATGATG | CTTAACTTCTACCAATTCCACAAAT | 60 |
| MTIF2 | 385 | AGGGATTAAATGTATTTTGGAAAGGTTTT | CCTAAAAAAAACCACAAATTAAACCA | 60 |
| MTIF3_Amplicon1 | 212 | TTTTGTTTTATTTGTGTTAGGTAGTAAG | AACCCCACTAAACTTTTCTAATTCC | 60 |
| MTIF3_Amplicon2 | 493 | AATTYAGAAAAGTTTAGTGGGGTTGG | TAAAACCAAATAAACAACAATTTCCC | 56 |
| MTIF3_Amplicon3 | 388 | AGTTTTTTTATTGTTGGGATTTAGTTT | ACCCCAACTTTATTTTACCTCC | 60 |
| OPA1_ Amplicon 1 | 482 | TGTTTAAGAAAGAAGGTAGGTAAATGTG | AAAATAACCCTCAACAACAAAAACA | 60 |
| OPA1_ Amplicon 2 | 209 | GTTGAGGGTTATTTTTTGGGTTATT | TAAAAATAAAACAACCCCACTCTCA | 60 |
| PARKIN | 212 | AGGAGAGGTTGTATTTGGTAGGTATTT | ACTCCCAACAAACCCTAAACC | 56 |
| PINK1_ Amplicon 1 | 600 | TAAAGTGTAAAGGGAAAGTTATTGT | CTCACCTAAATCTCCTAACAAACC | 60 |
| PINK1_ Amplicon 2 | 383 | GGTTTGTTAGGAGATTTAGGTGAG | AAAACTTTCCTTCTCCATAAATTAAAA | 60 |
| POLG1_ Amplicon 1 | 350 | AGGGGGTAGTTGGGTTTGTAATAGTAA | ACAACAACAACAACAACAACAACA | 58 |
| POLG1_ Amplicon 2 | 412 | AGGTTTTTTGATTGGAGAGGGAG | AAACCAATCCACCTACTCCTTAAA | 60 |
| POLG2 | 276 | AGAATAGGTATTTGTAGATTTTATGG | CAAACTAACAAAAAACCATCC | 60 |
| POLRMT_ Amplicon 1 | 478 | AGGTTTGTTTTAGAATTTGAGTTTTTGTTT | CTTATACAACCTCCTAACCCCAAAT | 60 |
| POLRMT_ Amplicon 2 | 153 | AGTTATATTTGGGGTTAGGAGGTTGTAT | TTTAACCTTTACCTCTTTACACCTAAAC | 58 |
| RAB32_ Amplicon 1 | 449 | AGGGGAGAGGAAGTTTAGTTGGGTT | TCACCAACACCTTAAAAAAATACTC | 60 |
| RAB32_ Amplicon 2 | 302 | AGGAGTATTTTTTTAAGGTGTTGGTGA | TCTTCTCCTAAAAATACCCAAACCAA | 60 |
| RHOT1 | 159 | AGGGTGTTTTTGGTGAGAGGAGTTTA | ACAAAAACAAAAAAAACAAAAACTCAA | 60 |
| RHOT2_ Amplicon 1 | 277 | TGGAGTTTTTTTGTTTTTTTAGAAG | AAATCCTATTATCCCTAAAACACCACT | 60 |
| RHOT2_ Amplicon 2 | 365 | TTTTAGGTTTTTAGATTAGGATTTGGA | AAACAAAACAAAAAAAACACCAAAC | 60 |
| TFAM_ Amplicon 1 | 172 | AGGGGGTTTTGGATGTAGGATT | CTATTAAACCACCTCTATCCCC | 58 |
| TFAM_ Amplicon 2 | 200 | AGGGGGATAGAGGTGGTTTAAT | CACTATAAAAAATCTACTAACATCC | 60 |
| TFB1M | 405 | GTTTTAAGTTTAGGAGGAAATTTTGTGA | CCAAAAAAAACCTAATCCACCC | 60 |
| TFB2M | 496 | TGGGATTTATATGTTTTTGTTTTTTAGG | AATCTATTTCCAACCCCACTC | 60 |
| TRAK1 | 260 | GAGGAGGAGTAAGAGAGGAAGTTTTAG | TAAATCAAAAACCAAAAAAAATCCC | 60 |
| TRAK2_ Amplicon 1 | 257 | AGGGATAGTTTATTTATTGGAGTGGTT | AAAAATAACTCTCCTTTAACTTCCCC | 58 |
| TRAK2_ Amplicon 2 | 232 | AGTGGTAGTTTTTATATAATTGGGGAA | TTAAAACTCTACACCAATCCCAAAC | 58 |
